# StackAge: an ensemble-based clock for precise quantification of biological age using multi-omics data

**DOI:** 10.1093/bib/bbag271

**Published:** 2026-05-31

**Authors:** Yingyi Jiang, Lei Jia, Yuan Fei, Xiaoguang Li, Xiaoqi Zheng, Yufang Qin

**Affiliations:** College of Information Technology, Shanghai Ocean University, 999 Hucheng Ring Road, Pudong New District, Shanghai 201306, China; Key Laboratory of Fisheries Information Ministry of Agriculture, 999 Hucheng Ring Road, Pudong New District, Shanghai 201306, China; Department of Biomaterials and Stem Cells, Suzhou Institute of Biomedical Engineering and Technology, Chinese Academy of Science, 88 Keling Road, Suzhou New District, Suzhou, Jiangsu Province, 215011, China; Center for Single-Cell Omics, School of Public Health, Shanghai Jiao Tong University School of Medicine, 1 Banxia Road, Pudong New District, Shanghai 200025, China; Center for Single-Cell Omics, School of Public Health, Shanghai Jiao Tong University School of Medicine, 1 Banxia Road, Pudong New District, Shanghai 200025, China; Center for Single-Cell Omics, School of Public Health, Shanghai Jiao Tong University School of Medicine, 1 Banxia Road, Pudong New District, Shanghai 200025, China; College of Information Technology, Shanghai Ocean University, 999 Hucheng Ring Road, Pudong New District, Shanghai 201306, China; Key Laboratory of Fisheries Information Ministry of Agriculture, 999 Hucheng Ring Road, Pudong New District, Shanghai 201306, China

**Keywords:** biological aging clock, multi-omics integration, ensemble learning, disease prediction, SHAP interpretability, UK biobank

## Abstract

Accurate quantification of biological age is essential for early risk stratification and intervention of chronic diseases. Here, we present StackAge, an ensemble-based biological aging clock that integrates large-scale plasma proteomic and metabolomic profiles from 30 376 participants in the UK Biobank. StackAge demonstrated high accuracy in age prediction (Pearson r ≈ 0.93 with chronological age) and substantially enhanced risk prediction for 12 chronic diseases, achieving AUCs exceeding 0.90 for type 2 diabetes, Alzheimer’s disease, and chronic kidney disease. Notably, the incorporation of estimated aging rates consistently improved disease prediction beyond conventional omics and demographic features. Feature interpretation and pathway enrichment analyses revealed that aging-associated biomarkers were enriched in inflammation, metabolic stress, and extracellular matrix remodeling pathways. Mediation analysis further indicated that modifiable lifestyle factors may accelerate biological aging, thereby increasing susceptibility to cardiovascular, neurological, immune, and musculoskeletal disorders. Together, these findings establish a robust multi-omics framework for quantifying individual aging trajectories and highlight biological age as a clinically actionable indicator for precision prevention and health management of age-related diseases.

## Introduction

Aging is a complex biological process characterized by progressive declines in cellular and molecular functions, leading to increased vulnerability to chronic diseases such as cardiovascular disorders, type 2 diabetes, neurodegenerative conditions, and cancer [1, 2]. Over the past decade, substantial efforts have focused on quantifying biological aging using molecular biomarkers to better understand aging mechanisms and their health consequences. Compared with biological age, biologically informed age estimates provide improved resolution for capturing inter-individual heterogeneity and have shown promise for disease risk prediction and personalized prevention strategies.

Biological aging clocks have consequently emerged as important computational tools for age estimation. Among available approaches, blood-based biomarkers are particularly advantageous because they can be collected with minimal invasiveness, exhibit high reproducibility, and reflect systemic physiological states across multiple organ systems [3, 4]. Early studies primarily relied on DNA methylation profiles as a surrogate indicator of biological age. For instance, Horvath and colleagues developed an epigenetic clock based on 353 CpG sites using elastic net regression [5, 6], while Hannum et al. constructed a blood-specific model using 71 CpG sites [7]. More recently, proteomic and metabolomic profiling has gained increasing attention because circulating proteins and metabolites provide direct and dynamic measurements of physiological and pathological processes. For example, Tanaka et al. analyzed 1301 plasma proteins to identify aging-related biomarkers [8], Argentieri et al. expanded this analysis to over 5000 proteins to build a proteomic aging clock predictive of mortality and common diseases [9], and Mutz et al. developed the MileAge metabolomic model using machine learning algorithms to predict health status and lifespan [10].

Despite these advances, current aging clocks exhibit several methodological limitations. Most models rely on a single molecular layer, either epigenomics, proteomics or metabolomics, therefore, may not fully capture the multidimensional and system-level nature of aging. Because aging involves coordinated interactions across immune, metabolic, and structural pathways, single-omics approaches may overlook cross-layer relationships and yield suboptimal predictive performance [11]. In additional, lifestyle behaviors such as diet, physical activity, smoking, and sleep are well-established determinants of aging and chronic disease onset [12]; however, their quantitative effects on biological aging, and their potential mediating roles in disease risk, remain insufficiently characterized within integrated computational frameworks.

To address these gaps, we developed a multi-omics biological aging clock that jointly integrates plasma proteomic and metabolomic data. Leveraging data from 30 376 participants in the UK Biobank, we propose StackAge, an ensemble-based framework that combines complementary linear and nonlinear learners to enhance predictive robustness and generalization while maintaining interpretability. Beyond chronological age estimation, we systematically evaluated the association between the derived aging rate and multiple chronic diseases and further investigate whether lifestyle factors influence disease risk through accelerated biological aging using mediation analysis. Collectively, this study provides an interpretable and scalable multi-omics framework for biological age modeling and offers a computational foundation for investigating aging-related disease mechanisms in large population cohorts.

## Materials and methods

### UK biobank cohort and participant selection

Data for this study were obtained from the UK Biobank (https://www.ukbiobank.ac.uk/), a large-scale prospective cohort comprising over 500 000 participants recruited across 22 assessment centers in England, Scotland, and Wales [13]. The UK Biobank provides extensive demographic, lifestyle, clinical, and multi-omics measurements for population-scale biomedical research. Participants were excluded if they had missing follow-up data, died prior to follow-up, presented with any chronic disease at baseline, or lacked essential demographic or lifestyle variables (*n* = 449 117). Individuals with more than 20% missing values in blood biochemistry or Olink assays were additionally removed (*n* = 22 682). After applying these criteria, 30 376 participants were retained for downstream analyses, with a median follow-up duration of 11.7 years.

Participants were categorized into a disease-free (healthy) cohort (*n* = 16 078) and a disease cohort (*n* = 14 298). To strictly prevent information leakage, the healthy cohort was randomly partitioned into a training set (80%) and an independent test set (20%) using a fixed random seed. All feature selection, model training, and hyperparameter optimization procedures were conducted within the training set. The independent test set was reserved solely for final evaluation.

### Data collection and preprocessing

For each participant, demographic (e.g. age, sex, BMI, and smoking status), lifestyle (e.g. diet, physical activity, sleep duration, alcohol consumption), proteomic, and metabolomic variables were collected. Plasma proteomics comprised 2923 proteins quantified using the Olink Explore platform based on proximity extension assay technology, whereas metabolomics included 251 circulating metabolites measured by nuclear magnetic resonance spectroscopy using the Nightingale Health platform. Missing demographic variables (~2%) were imputed using subgroup means for continuous variables and modal categories for categorical variables. For proteomic and metabolomic features, missing values (~3%) were set to zeros following standard platform-specific preprocessing protocols. Continuous variables were standardized using Z-score normalization, and categorical variables (e.g. sex, smoking status, and alcohol intake) were one-hot encoded prior to model training.

Chronological age was calculated as the decimal difference between the date of birth and the date of plasma sampling (field ID 21842), using 365.25 days per year to account for leap years, rather than the integer-based recruitment age (field ID 21022). Disease outcomes were defined using hospital inpatient records from the UK Biobank based on ICD-10 diagnosis codes (Supplemental Table 1).

### Feature selection

Feature selection was conducted within the training subset of the healthy cohort. Initially, a baseline LightGBM regressor was trained using all 3174 proteomic and metabolomic features to predict chronological age. SHapley Additive exPlanations (SHAP) values were subsequently computed from this fitted model using TreeExplainer to quantify each feature’s contribution to the prediction. These values represent the additive contributions relative to the model’s expected output: positive SHAP values indicate that a feature increases the predicted age (i.e. contributes to accelerated biological aging), whereas negative values indicate contributions toward a younger predicted age. Feature importance was ranked based on the mean absolute SHAP value across all training samples (mean |SHAP|). To determine the optimal number of features, predictive performance was further evaluated across various subset sizes (ranging from the top 50 to 800 features). Performance improved substantially up to approximately 300 features and plateaued thereafter. Consequently, the top 300 features were selected to balance accuracy, model complexity, and interpretability (Supplemental Table 2).

### Construction of the StackAge model

Using the selected features, we developed an ensemble-based biological age prediction framework termed StackAge. The framework utilizes a stacking architecture that integrates three complementary base learners (Linear Regression, ElasticNet, and LightGBM), whose predictions are combined using an XGBoost meta-learner. This design leverages both linear and nonlinear modeling capacities to enhance generalization performance. Five-fold cross-validation was used to generate out-of-fold predictions, whereby base learners were trained on four folds and evaluated on the held-out fold. After cross-validation, the final StackAge model was refitted on the complete training set and evaluated exclusively on the independent test set to obtain an unbiased estimate of generalization performance.

### Disease risk prediction based on aging rate

To evaluate the association between biological aging and disease onset, we constructed four predictive models for 12 age-related diseases using a stepwise feature inclusion strategy:

(1) Model 1: Proteomics + metabolomics + demographics (age, sex, BMI) + aging rate
(2) Model 2: Identical to Model 1 but excluding the aging rate
(3) Model 3: Proteomic and metabolomic features only
(4) Model 4: Proteomic features only

All models were implemented using the LightGBM framework for binary classification to predict incident disease risk at 5-, 10-, and 15-year follow-up intervals. A fixed random seed and standardized time-to-event definitions were used to ensure methodological consistency.

To further investigate the biological relevance of aging-associated biomarkers, we conducted an independent differential expression analysis of the 286 proteins contributing to aging rate estimation. For each protein, the fold change (FC) between disease and matched healthy samples was calculated as the ratio of mean protein abundances, while statistical significance (*p*) was assessed using two-sided Welch’s *t-*tests. To prioritize proteins exhibiting both substantial effect sizes and high statistical significance, we utilized a composite ranking score defined as |log_2_FC| × (−log_10_  *p*). Based on this metric, the top 10 upregulated and top 5 downregulated proteins were identified as candidate disease-associated biomarkers for each clinical endpoint.

### Mediation analysis

To examine whether biological aging mediates the relationship between lifestyle behaviors and disease risk, we performed mediation analysis using structural equation modeling (SEM) [14]. All variables were normalized using MinMaxScaler [15] to ensure comparability across features, and two hierarchical models were constructed, including a baseline model including only the direct path from lifestyle behavior to disease risk (e.g. Smoking_Score → Disease) and an extended mediation model incorporating aging rate as an intermediate variable (Smoking_Score → Aging_Rate → Disease). Model parameters were estimated using the maximum likelihood estimation, and statistical significance of path coefficients was evaluated based on their standard errors and corresponding *z*-statistics [16], with a predefined threshold of *P* < .1. Indirect (mediation) effects were assessed using the Sobel test [17], which evaluates whether the product of path coefficients significantly differs from zero, and the proportion mediated, defined as the ratio of indirect to total effects, was additionally calculated to quantify the extent to which aging rate explained the association between lifestyle factors and disease outcomes.

### Statistical analysis

Differences in aging rate across demographic subgroups were systematically evaluated. Race was harmonized into five categories, and overall group differences were assessed using one-way analysis of variance [18], while sex differences were evaluated using independent-sample t-tests, with outlier-adjusted estimates to reduce the influence of extreme values. Time-to-event associations between aging rate and incident diseases were assessed using Kaplan–Meier survival analysis [19]. For lifestyle comparisons, differences in predicted biological age between healthy and unhealthy groups were quantified using estimated mean differences (Δ, years), 95% confidence intervals, and standardized effect sizes (Cohen’s d). All statistical analyses were performed using Python-based scientific computing libraries unless otherwise specified.

## Results

### Study design and analytical framework

**Figure 1:**
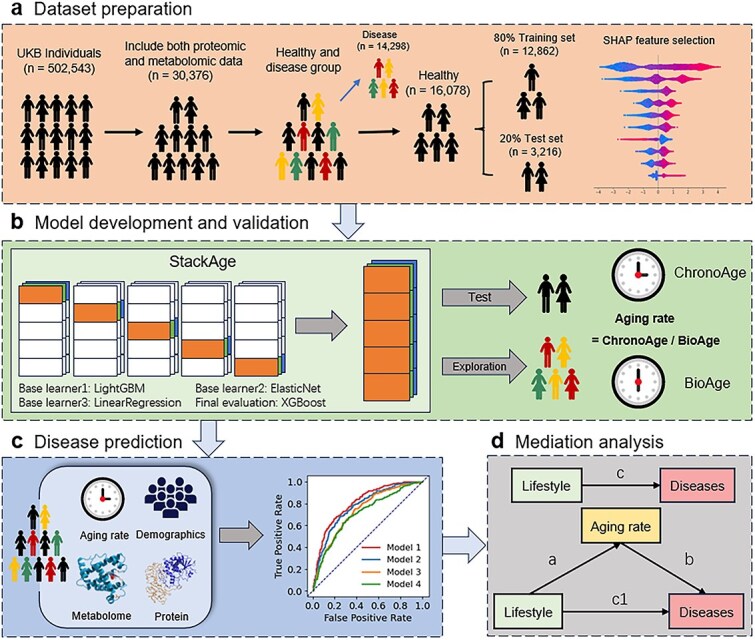
Overview of the study design and framework of StackAge. (a) Data preprocessing and feature selection. Multi-omics profiles (2923 proteins, 251 metabolites) from 30 376 UK Biobank participants were analyzed. Based on disease status, participants were divided into a healthy (*n* = 16 078) and disease (n = 14 298) groups. SHAP values ranked 3175 features, and the top 300 were used for model development. (b) StackAge, a meta-learning age prediction model by integrating linear regression, LightGBM, and ElasticNet as base learners. Aging rate for each participant was calculated as the ratio of predicted biological age to chronological age. (c) Aging rate, together with omics and demographic variables, was used to predict 5-, 10-, and 15-year risks of 12 chronic diseases. (d) SEM assessed aging rate as a mediator linking lifestyle factors to disease risk.

We established a multi-omics analytical framework to construct a biological age prediction model and to systematically evaluate the relationship between aging rate and various age-related diseases (Fig.  1). From the UK Biobank cohort of 502 543 participants, 30 376 individuals with available plasma proteomic and metabolomic measurements were retained after rigorous quality control (see Methods). Based on baseline disease status, participants were stratified into a healthy cohort (*n* = 16 078) and a disease cohort (*n* = 14 298). The healthy cohort was randomly divided into training (80%) and independent test (20%) sets. Within the training set, a baseline LightGBM regressor was initially trained using all 3174 proteomic and metabolomic features to predict chronological age. SHAP values derived from this fitted model were subsequently used to rank features and identify biomarkers most informative for biological aging (Fig.  1a).

A biological age prediction model was subsequently developed using data from the healthy cohort. We implemented an ensemble learning framework, termed StackAge, which integrates LightGBM, ElasticNet, and linear regression as base learners [20]. Model performance was evaluated via five-fold cross-validation, and the trained model was subsequently applied to the disease group for out-of-sample prediction. For each individual, the aging rate was defined as the ratio of predicted biological age to chronological age, serving as a quantitative metric for biological age acceleration or deceleration (Fig.  1b).

To further assess the prognostic value of biological aging, we constructed four disease prediction models using different combinations of omics, demographic variables, and aging rate to estimate the risk of incident diseases across 5-, 10-, and 15-year follow-up intervals (Fig.  1c). Finally, to investigate the potential mechanisms linking lifestyle behaviors to disease outcomes, mediation analysis was performed by jointly modeling lifestyle behaviors, aging rate, and disease risk within an integrated framework (Fig.  1d).

### Feature selection and model evaluation

**Figure 2:**
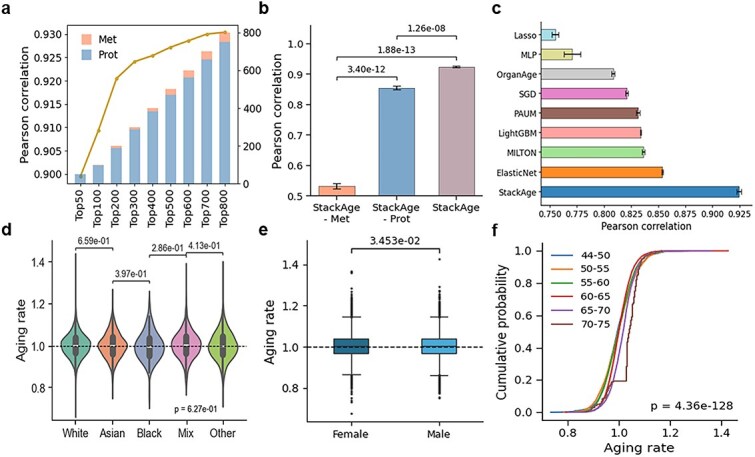
Performance evaluation and population-level differences in aging rate. (a) Contributions of proteomic and metabolomic features to biological age prediction (top 50–800 features ranked by SHAP value). (b) Performance comparison of models trained with different omics inputs. The integrated StackAge model was constructed using SHAP-selected features from both proteomic and metabolomic data (286 proteins +14 metabolites). The proteomic-only model was trained using the top 300 SHAP-ranked proteins, while the metabolomic-only model was trained using all available metabolites (*n* = 251), as the metabolite panel size was limited. This design enables a fair comparison of the relative and complementary contributions of each omics modality to biological age prediction. (c) Comparison of StackAge with published aging clocks and conventional machine learning and deep learning baseline models using proteomic and metabolomic inputs. (d, e) Distributions of predicted aging rate across race (d) and sex (e). (f) Cumulative distributions of predicted aging rate by StackAge across six chronological age groups (44–75 years).

We employed SHAP values to rank the importance of all 3174 omics-derived features (2923 proteins and 251 metabolites) based on the training dataset (Supplemental Fig. 1). To balance predictive accuracy and model complexity, we selected the top 300 ranked features for subsequent model construction. Using this reduced feature set, StackAge achieved a strong correlation between predicted biological age and chronological age in the independent test set (Pearson r = 0.924; Fig.  2a).

To assess the contribution of each omics modality, we compared model performance using single-omics and integrated multi-omics inputs (Fig.  2b). When training solely on the 251 metabolomic features, the model achieved a moderate Pearson correlation of *r* = 0.53. In contrast, the proteomic-only model using the top 300 proteins achieved a substantially higher correlation (*r* = 0.86). Our final StackAge model by integrating both proteomic and metabolomic data (286 proteins +14 metabolites) further improved performance (*r* = 0.93). These results underscore the superiority of the multi-omics integration over single-omics models, and highlighted the predominant predictive contribution of the proteomic layer.

We further benchmarked StackAge against several recently published aging clocks, including Milton [21], Paum [9], and OrganAge [22], as well as several conventional machine learning (Lasso, SGD, LightGBM, ElasticNet) and deep learning baselines (MLP). To ensure a fair and consistent evaluation, all published aging clocks were reimplemented and retrained using the identical training and independent test splits as StackAge, without pretrained weights or external parameters. Across this comprehensive comparison, StackAge consistently achieved superior predictive performance (Fig.  2c), demonstrating improved robustness and generalization. To further quantify the contribution of individual base learners, we conducted a systematic component ablation analysis. The full StackAge ensemble achieved the best overall performance (Pearson *r* = 0.924, RMSE = 3.081, MAE = 2.42, R^2^ = 0.854), whereas exclusion of any component resulted in consistent performance degradation (Supplemental Table 3). The largest decline was observed when ElasticNet was removed, indicating that regularized linear modeling contributes substantially to predictive performance, while the remaining learners provide complementary information.

### Population heterogeneity of aging rate

We next characterized inter-individual variability in predicted aging rates across demographic strata. The distribution of aging rates was comparable across racial groups (Fig.  2d), supporting cross-population robustness of the model. In contrast, a modest but statistically significant sex difference was observed, with males exhibiting slightly higher median aging rates than females (Fig.  2e). Sensitivity analyses confirmed that aging rates were orthogonal to chronological age (Pearson *r* = 0.003), and the mean age difference between sexes was negligible (0.36 years). Furthermore, the sex effect remained significant after age adjustment in an ANCOVA, indicating that the observed sex difference was not driven by age-related bias (Supplemental Table 4). When stratified by chronological age (44–75 years), aging rate showed a gradual upward trend with increasing age, with more pronounced elevation after age 65 (Fig.  2f). This suggests that the aging rate effectively captures systematic age-related shifts in biological age while preserving individual-level heterogeneity.

### Prognostic value of aging rate for age-related diseases

**Figure 3:**
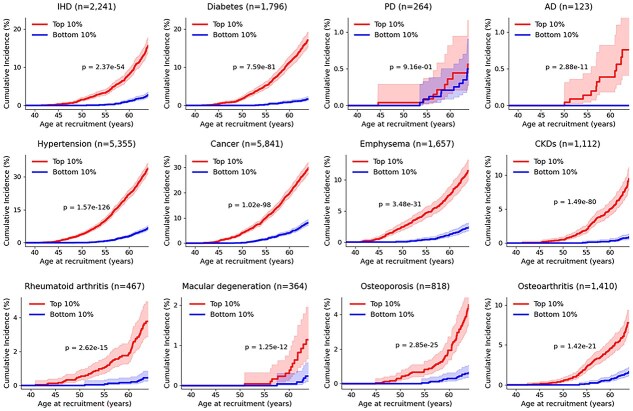
Cumulative incidence of 12 chronic diseases by aging rate strata. Kaplan–Meier curves show age-specific incidence for ischemic heart disease, type 2 diabetes, Parkinson’s disease, Alzheimer’s disease, hypertension, cancer, emphysema, CKD, rheumatoid arthritis, macular degeneration, osteoporosis, and osteoarthritis. Participants in the top 10% aging rate (red) were compared with the bottom 10% (blue); shaded areas indicate 95% CIs.

We next evaluated the prognostic value of aging rate for 12 representative aging-related diseases (Fig.  3). To this end, participants were stratified into the top 10% (high aging rate) and bottom 10% (low aging rate). Across all 12 diseases, the high aging rate group consistently exhibited higher cumulative incidence, with particularly pronounced separation for common chronic conditions such as type 2 diabetes, hypertension, coronary artery disease, chronic kidney disease (CKD), and rheumatoid arthritis. For several neurodegenerative or ophthalmic conditions, including Alzheimer’s disease and macular degeneration, similar trends were observed, although the 95% confidence intervals partially overlapped, likely reflecting smaller event counts and lower incidence rates before age 65. Overall, these findings support a robust association between accelerated biological aging and increased risk of multiple chronic diseases.

To further assess the robustness of these associations, we conducted several sensitivity analyses. First, restricting the cohort to participants diagnosed with only a single disease yielded consistent separation between the top-10% and bottom-10% aging-rate groups for hypertension and cancer, the two endpoints with sufficient case numbers after restriction (Supplemental Fig. 2). In addition, retraining StackAge after excluding disease-specific upregulated proteins resulted in only minimal reductions in predictive performance (Supplemental Table 5), indicating that model captures systemic aging-related signals rather than disease-specific biomarkers. Similar cumulative incidence patterns were also observed when residual-based age acceleration was used as an alternative aging definition (Supplemental Fig. 3). Collectively, these analyses confirm that the observed associations are robust across alternative cohort definitions, feature sets, and aging metrics.

### Disease risk prediction using aging rate and multi-omics features

**Figure 4:**
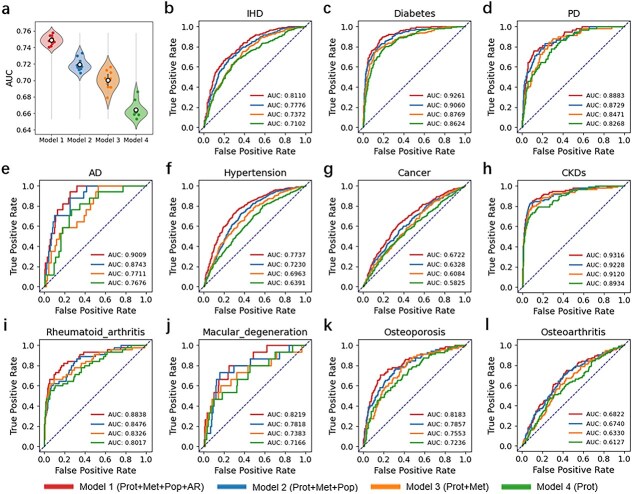
Predictive performance of aging rate and multi-omics feature for disease risk prediction (5-year). (a) Comparison of four predictive models with different combinations of input features: Model 1 (proteomics + metabolomics + demographics + aging rate), Model 2 (proteomics + metabolomics + demographics), Model 3 (proteomics + metabolomics), and Model 4 (proteomics only). Each dot represents the AUC from one fold of 10-fold stratified cross-validation for disease risk prediction. The hollow circle indicates the mean AUC, and the violin shows the distribution of AUC values across folds. (b–l) AUCs (0.54–0.93) for 12 chronic diseases.

To assess the predictive utility of aging rate and multi-omics features for disease risk stratification, we developed four classification models using different combinations of input variables (Fig.  4). Model 1 incorporated proteomic biomarkers, metabolites, demographic characteristics, and aging rate; Model 2 included the same features but excluded aging rate; Model 3 used proteomic and metabolomic features only; and Model 4 relied solely on proteomic features. Across all prediction horizons (5-year, 10-year and 15-year follow-up), Model 1 consistently achieved the best performance, indicating that inclusion of aging rate provides complementary predictive information beyond raw omics and demographic variables. Notably, incorporation of the aging rate resulted in a 2%–4% improvement in AUC, demonstrating its incremental value for disease risk prediction. Among the three time horizons, the highest predictive accuracy was observed for 5-year risk prediction (Supplemental Fig. 4). Comparable predictive accuracy was also obtained when residual-based age acceleration was used in place of the ratio-based aging rate, indicating that the predictive utility of biological aging remained robust across alternative aging definitions (Supplemental Fig. 5).

### Functional interpretation of aging-associated proteins

**Figure 5:**
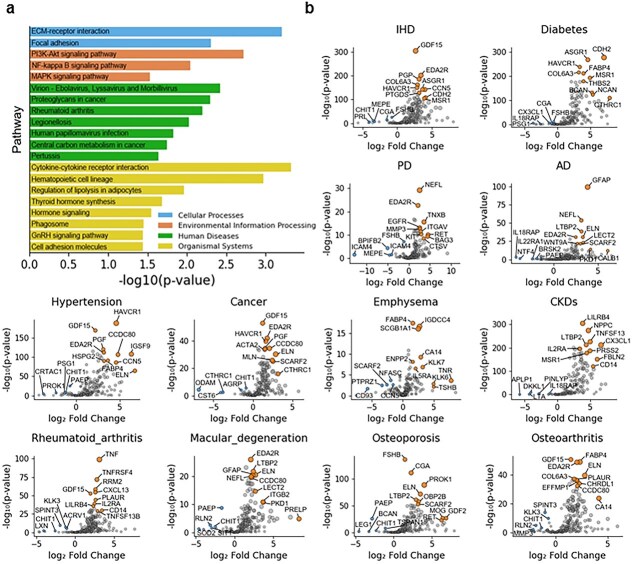
Functional enrichment and differential expression of aging-related proteins. (a) KEGG analysis of 286 proteins identified in the model. (b) Volcano plot showing differential protein expression between disease cases and healthy controls based on log₂ fold change and Welch’s *t-*test *P* values. The top 10 upregulated (orange) and top 5 downregulated (blue) proteins ranked by |log₂FC| × (−log_10_  *p*) are highlighted.

To explore the biological mechanisms underlying age-related diseases risk, we performed KEGG pathway enrichment analysis on the 286 proteins prioritized by SHAP importance (Supplemental Table 6). Several pathways with established roles in aging and age-associated diseases were significantly enriched, including cytokine–cytokine receptor interaction, PI3K-Akt signaling, ECM–receptor interaction, NF-kappa B signaling, and MAPK signaling (Fig.  5a). These pathways involve key proteins such as GDF15, TNF, EDA2R, TNFRSF1B, THBS1, NFKB1, and RELA, which collectively regulate inflammation, immune responses, cellular stress, and metabolic homeostasis [23]. Independent differential expression analysis across 12 diseases (Fig.  5b) further demonstrated consistent upregulation of these proteins in conditions including rheumatoid arthritis, cancer, CKD, and diabetes [24], while disease-specific markers such as NEFL, HAVCR1, and GFAP, were additionally elevated in neurodegenerative and autoimmune disorders [25]. Notably, the disease-associated proteins identified across all endpoints were predominantly linked to inflammation, extracellular matrix remodeling, metabolic stress, and tissue injury, rather than canonical cell-cycle regulators, suggesting that the aging rate signal reflects systemic physiological decline rather than alterations in cellular proliferation. Collectively, these results suggest that aging-associated proteins converge on shared inflammatory and stress-related pathways that contributing to disease susceptibility.

### Mediation role of aging rate in disease incidence

**Figure 6:**
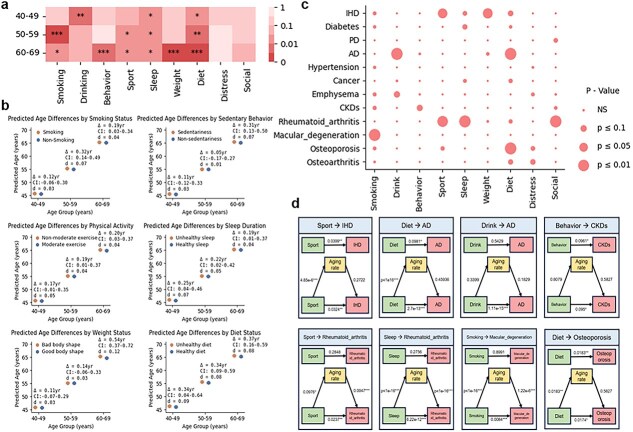
Associations of lifestyle, biological aging, and chronic disease. (a) Heatmap summarizing the statistical significance of the contrast in predicted biological age between the “healthy” and “unhealthy” groups for each lifestyle factor within three chronological age strata (40–49, 50–59, and 60–69 years). Cell colors encode the two-sided *P* values (darker color indicates smaller *P* values), and asterisks denote significance levels (^*^  *P* ≤ .05; ^**^  *P* ≤ .01; ^***^  *P* ≤ .001). (b) Estimated differences in predicted biological age between healthy and unhealthy lifestyle groups across three chronological age strata. Points indicate group means, horizontal lines represent 95% confidence intervals of the mean difference (Δ, years), and annotations report Δ and Cohen’s d effect sizes for improved interpretability. (c) Mediation matrix illustrates indirect effects of lifestyle on 12 diseases via aging rate. (d) Path diagrams highlight eight significant life style → aging rate → disease mediation pathways.

We next examined the impact of modifiable lifestyle factors on biological aging and subsequent disease risk. Nine lifestyle-related variables were considered, including smoking status, alcohol consumption, sedentary behavior, physical activity, sleep duration, weight status, dietary quality, depressive mood, and social engagement. For each factor, participants were categorized into “healthy” and “unhealthy” groups, and predicted biological ages were compared across three chronological age strata (40–49, 50–59, and 60–69 years; Fig.  6a). Our results indicated that several adverse lifestyle factors were consistently associated with older predicted biological age. For instance, smokers exhibited higher predicted ages than non-smokers, with mean differences ranging from 0.12 to 0.32 years across age groups (Cohen’s d = 0.03–0.07, Fig.  6b). Physical inactivity, short sleep duration, and unhealthy dietary patterns showed similar trends, with modest but consistent effect sizes. In contrast, sedentary behavior and psychosocial distress demonstrated weaker or negligible associations in some age strata, as indicated by confidence intervals overlapping zero. Although the absolute differences were small, the consistent direction of effects suggests that unfavorable lifestyle behaviors may cumulatively contribute to accelerated biological aging.

To determine whether these lifestyle-associated changes translate into downstream disease susceptibility through biological aging, we next conducted mediation analyses using aging rate as the mediator. Treating the nine lifestyle factors as independent variables and 12 age-related chronic diseases as outcomes, we identified six significant mediation pathways (*P* < .01; Fig.  6c, and  d). Specifically, physical inactivity was associated with elevated aging rate and increased cardiovascular disease risk; frequent alcohol consumption accelerated biological aging and was linked to higher Alzheimer’s disease risk; prolonged sedentary behavior was associated with accelerated aging and increased risk of CKD; and smoking was related to higher aging rates and greater risk of age-related macular degeneration. To further provide molecular resolution beyond this composite metric, we also conducted protein-level mediation analyses by replacing aging rate with representative disease-associated proteins (GDF15, GFAP, and EDA2R). Consistent indirect effects were observed, supporting the involvement of specific molecular pathways (Supplemental Fig. 6). Collectively, these findings support aging rate as a potential mechanistic link between lifestyle behaviors and disease susceptibility.

## Discussion and conclusion

This study leveraged data from UK Biobank to develop an ensemble-based framework that integrates multi-omics measurements for accurate quantification of biological age. The derived aging rate demonstrated consistent utility for disease risk stratification. Longitudinal analyses further indicated that individuals with accelerated aging rates were more likely to develop chronic conditions, including CKD and type 2 diabetes, within a 5-year follow-up period. Our mediation analysis further provided mechanistic insights, suggesting that biological aging may act as an intermediate pathway linking lifestyle behaviors to disease outcomes, such that smoking and unhealthy dietary patterns were associated with accelerated biological aging, which in turn corresponded to increased disease susceptibility. Together, these findings support biological aging as a potential mediator connecting modifiable risk factors with long-term health trajectories.

At the molecular level, KEGG enrichment analysis of aging-associated proteins revealed significant involvement of immune regulation, inflammatory signaling, and extracellular matrix remodeling pathways. Several representative biomarkers, including GDF15 and ASGR1, were enriched in processes related to inflammation, lipid metabolism, and mitochondrial dysfunction. Notably, these molecular alterations were detectable prior to clinical disease onset, suggesting shared early mechanisms between accelerated biological aging and chronic disease development [26–28]. Collectively, these observations provide biological support for the aging rate as a systems-level indicator of physiological decline and disease vulnerability.

Despite these strengths, several limitations should be acknowledged. First, the UK Biobank cohort consists predominantly of individuals of European ancestry, which may limit the generalizability of our findings to other populations. Second, the cross-sectional design of the proteomic and metabolomic measurements precludes the direct assessment of intra-individual aging dynamics, and longitudinal multi-omics profiling will be essential to elucidate temporal trajectories and causal relationships. Third, although key demographic variables were included, several established covariates in aging and disease risk studies, such as socioeconomic status, medication use, and pre-existing comorbidities, were not explicitly modeled. In addition, the current targeted omics platforms provide limited molecular coverage and may overlook organ-specific aging heterogeneity [29]. Future studies may address these limitations by expanding molecular profiling, incorporating tissue- or organ-specific biomarkers and imaging modalities [22, 30–32], and integrating mechanistic or causal modeling approaches to enable *in silico* simulation of protein or metabolite perturbations, thereby providing deeper insights into the biological drivers of aging. Embedding such extended aging metrics into personalized health monitoring systems may further facilitate continuous risk assessment and early intervention [33, 34].

**Key Points**

- StackAge is an ensemble-based biological aging clock that integrates large-scale plasma proteomic and metabolomic data from the UK Biobank to estimate individual biological age and aging rate with high accuracy.
- By leveraging a stacking framework that combines linear regression, ElasticNet, LightGBM, and an XGBoost meta-learner, StackAge achieves strong predictive performance (r ≈ 0.93 with chronological age) and outperforms existing single-omics and conventional aging clocks.
- The derived aging rate shows consistent prognostic value across 12 age-related chronic diseases and improves disease risk prediction over 5-, 10-, and 15-year follow-up horizons.
- Mediation analysis indicates that modifiable lifestyle factors partly influence disease risk partly through accelerated biological aging, supporting aging rate as a potential mechanistic link between behavior and disease susceptibility.

## Supplementary Material

Supplementary_Material_bbag271

## Data Availability

The original individual-level data used in this study are from UK Biobank and cannot be publicly shared due to data use restrictions. Access to the UK Biobank data can be granted by the UK Biobank Access Committee following a formal application process (https://www.ukbiobank.ac.uk/). This research was conducted under UK Biobank Application Number 116409. The analysis code and derived data generated for this study (including the age clock model) are publicly available in the following GitHub repository: https://github.com/PeterJiang122/JYY_AgeClock_20260205/releases/.
