# Supplementary material for: StackAge: an ensemble-based clock for precise quantification of biological age using multi-omics data
Source: Brief Bioinform. 2026 May 31;27(3):bbag271. doi: 10.1093/bib/bbag271 (PMC13222527; doi:10.1093/bib/bbag271)
Supplement: Supplementary_Material_bbag271 [file supplementary_material_bbag271.zip › Supplemental_Figures_bbag271.docx]

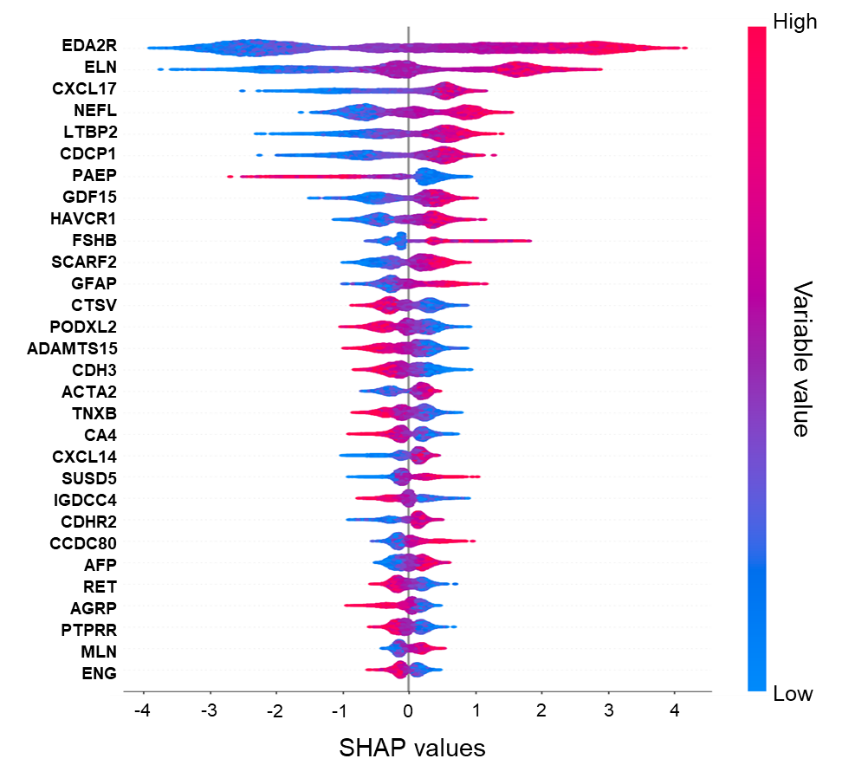


**Supplemental Figure 1:** Feature importance analysis based on SHAP values. The SHAP summary plot ranks the top 30 biomarkers by their contribution to the prediction model. The y-axis shows the biomarker names, and the x-axis displays the SHAP values, which indicate the magnitude and direction of each biomarker's impact on the model's output. Each point on the plot represents a sample, with its color signifying the biomarker's concentration (red for high, blue for low). The plot's point density shows the distribution of effects, revealing the consistency and variability of each biomarker's influence.


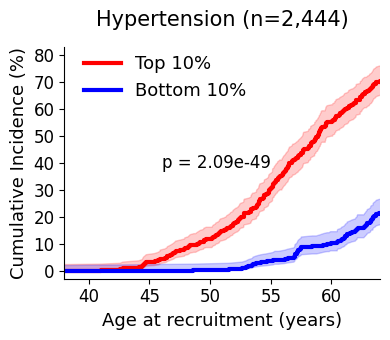

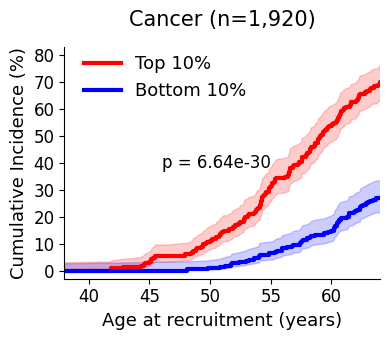


**Supplemental Figure 2:** Sensitivity analysis using participants with single-disease diagnoses only.


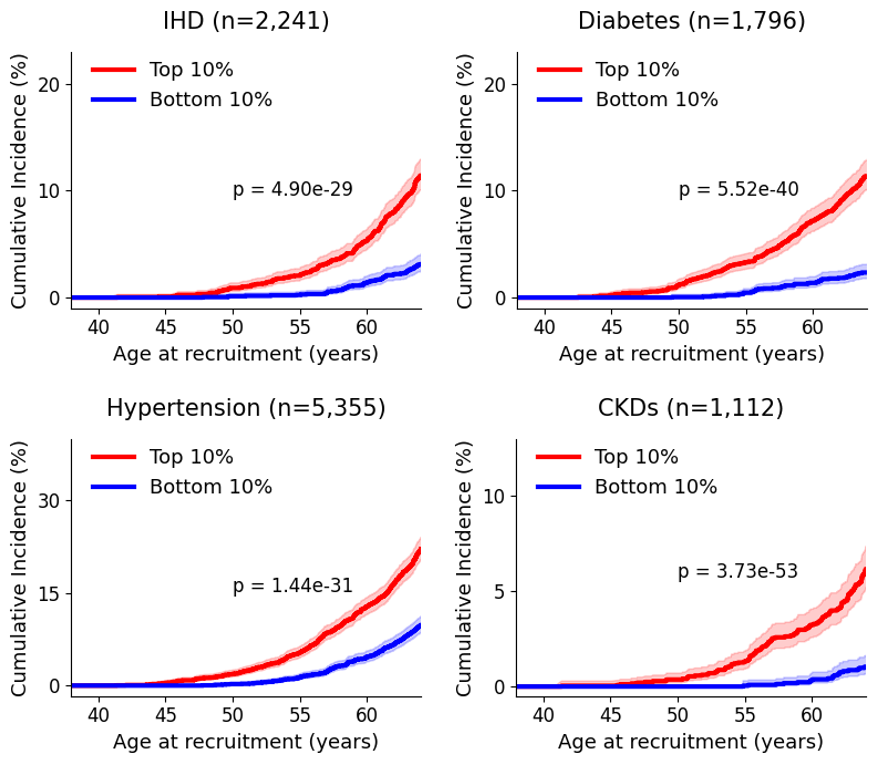


**Supplemental Figure 3:** Sensitivity analysis using residual-based age acceleration for cumulative incidence of age-related diseases.


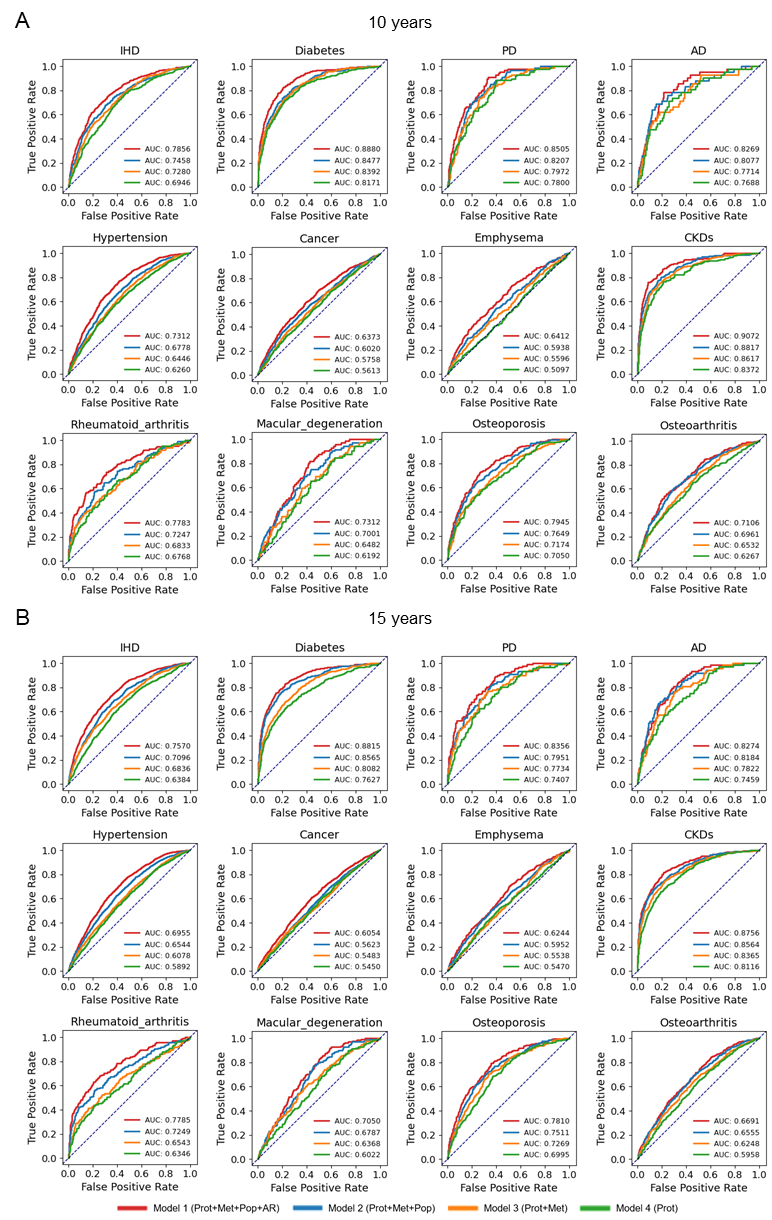


**Supplemental Figure 4:** Performance evaluation of 10- and 15-year disease risk prediction using LightGBM models based on plasma proteomics, metabolomics, and aging rate features. **(A, B)** Panels a and b display the performance of LightGBM models in predicting the 10- and 15-year incidence risk for 12 chronic diseases. The models used plasma proteomics, metabolomics, and aging rate as features. The Area Under the Curve (AUC) values, ranging from 0.5412 to 0.9316, showed significant variability in predictive performance across diseases like hypertension, diabetes, and cancer. The figure also includes the false positive rate, providing a basis for future model refinement.


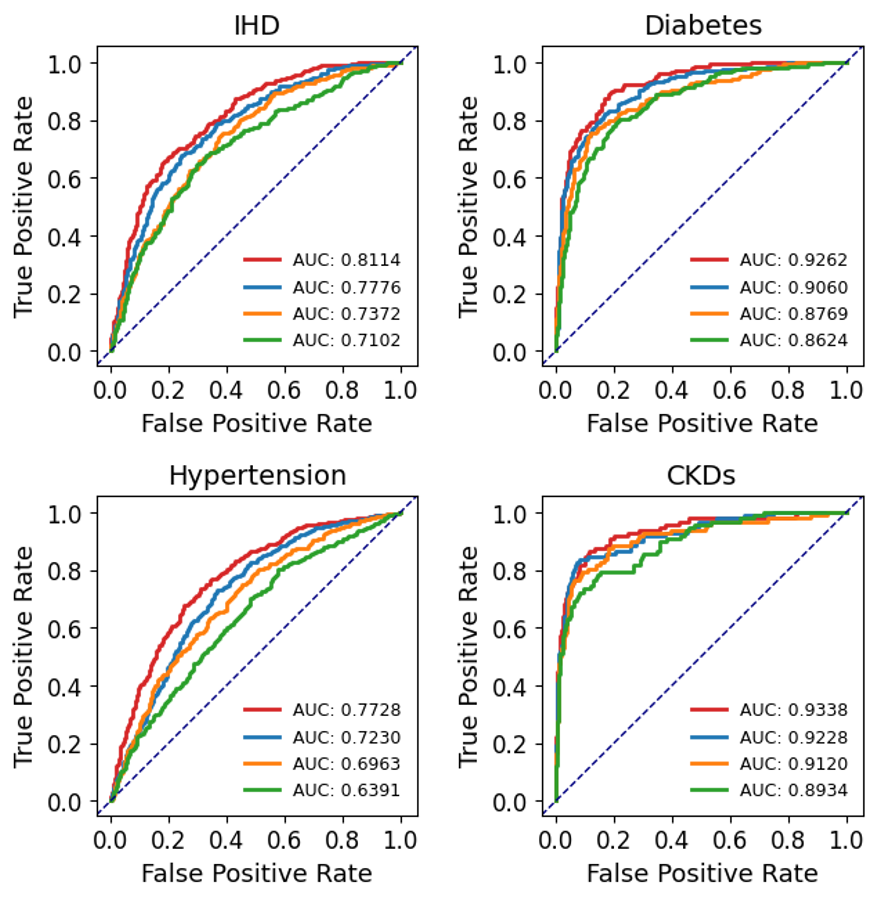


**Supplemental Figure 5:** Sensitivity analysis of disease risk prediction using residual-based age acceleration.


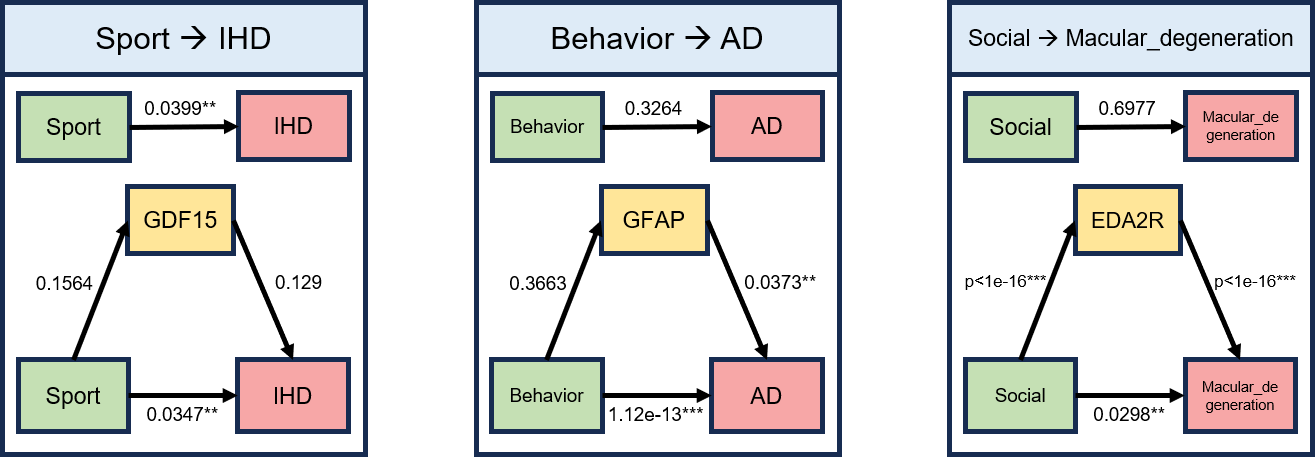


**Supplemental Figure 6: Protein-level mediation analyses linking lifestyle factors to disease risk.** Representative disease-related proteins (GDF15, GFAP, and EDA2R) were used as mediators to replace aging rate, demonstrating that specific molecular changes partially explain the links between lifestyle factors and disease risk.
