# Supplementary material for: StackAge: an ensemble-based clock for precise quantification of biological age using multi-omics data
Source: Brief Bioinform. 2026 May 31;27(3):bbag271. doi: 10.1093/bib/bbag271 (PMC13222527; doi:10.1093/bib/bbag271)
Supplement: Supplementary_Material_bbag271 [file supplementary_material_bbag271.zip › Supplemental_Tables_bbag271.docx]

Supplemental Table 1: ICD-10 codes used to define disease outcomes

| Disease | ICD-10 codes | Description |
| --- | --- | --- |
| IHD | I20–I25 | Ischemic heart diseases |
| Diabetes | E10–E14 | Type 1 and type 2 diabetes |
| PD | G20 | Parkinson’s disease |
| AD | G30 | Alzheimer’s disease |
| Hypertension | I10–I15 | Hypertensive diseases |
| Cancer | C00–C97 | Malignant neoplasms |
| Emphysema | J43 | Emphysema |
| CKDs | N18 (with N03, N11) | Chronic kidney disease |
| Rheumatoid arthritis | M05–M06 | Rheumatoid arthritis |
| Macular degeneration | H35.3 | Age-related macular degeneration |
| Osteoporosis | M80–M82 | Osteoporosis |
| Osteoarthritis | M00、M08、M09、M13 | Osteoarthritis |

Supplemental Table 2: List of the top 300 selected omics features ranked by mean |SHAP| importance

| Rank | Type | Feature_Name | Importance | Rank | Type | Feature_Name | Importance |
| --- | --- | --- | --- | --- | --- | --- | --- |
| 1 | Protein | EDA2R | 1.877140 | 144 | Protein | EPHB6 | 0.017118 |
| 2 | Protein | ELN | 1.177364 | 145 | Protein | NOTCH3 | 0.017064 |
| 3 | Protein | CXCL17 | 0.629895 | 146 | Protein | OBP2B | 0.016807 |
| 4 | Protein | NEFL | 0.628135 | 147 | Protein | HAGH | 0.016690 |
| 5 | Protein | LTBP2 | 0.583965 | 148 | Protein | SCGB1A1 | 0.016311 |
| 6 | Protein | CDCP1 | 0.581551 | 149 | Protein | ODAM | 0.016249 |
| 7 | Protein | PAEP | 0.444085 | 150 | Protein | KLK10 | 0.016229 |
| 8 | Protein | GDF15 | 0.420543 | 151 | Protein | KLK8 | 0.016087 |
| 9 | Protein | HAVCR1 | 0.375437 | 152 | Protein | SCG3 | 0.015942 |
| 10 | Protein | FSHB | 0.332436 | 153 | Protein | CHAD | 0.015842 |
| 11 | Protein | SCARF2 | 0.330320 | 154 | Protein | CXCL12 | 0.015014 |
| 12 | Protein | GFAP | 0.305957 | 155 | Protein | C19orf12 | 0.015002 |
| 13 | Protein | CTSV | 0.286075 | 156 | Protein | DMP1 | 0.014785 |
| 14 | Protein | PODXL2 | 0.273579 | 157 | Protein | IL1A | 0.014669 |
| 15 | Protein | ADAMTS15 | 0.263464 | 158 | Protein | VAMP5 | 0.014657 |
| 16 | Protein | CDH3 | 0.246095 | 159 | Protein | CTSF | 0.014056 |
| 17 | Protein | ACTA2 | 0.236931 | 160 | Protein | IL5RA | 0.013978 |
| 18 | Protein | TNXB | 0.229154 | 161 | Protein | SIGLEC7 | 0.013910 |
| 19 | Protein | CA4 | 0.208365 | 162 | Protein | PGF | 0.013768 |
| 20 | Protein | CXCL14 | 0.193612 | 163 | Protein | TAFA5 | 0.013564 |
| 21 | Protein | SUSD5 | 0.192327 | 164 | Protein | LAG3 | 0.013556 |
| 22 | Protein | IGDCC4 | 0.188461 | 165 | Protein | PTX3 | 0.013488 |
| 23 | Protein | CDHR2 | 0.186960 | 166 | Protein | CLEC1A | 0.013480 |
| 24 | Protein | CCDC80 | 0.182561 | 167 | Protein | CRLF1 | 0.013451 |
| 25 | Protein | AFP | 0.172367 | 168 | Protein | RGMA | 0.013405 |
| 26 | Protein | RET | 0.171589 | 169 | Protein | CKAP4 | 0.013324 |
| 27 | Protein | AGRP | 0.168857 | 170 | Protein | HSPG2 | 0.013023 |
| 28 | Protein | PTPRR | 0.158390 | 171 | Protein | PIGR | 0.013010 |
| 29 | Protein | MLN | 0.150769 | 172 | Protein | CNTN2 | 0.013009 |
| 30 | Protein | ENG | 0.149076 | 173 | Protein | DUSP13 | 0.012913 |
| 31 | Protein | KLK3 | 0.142090 | 174 | Protein | GPC1 | 0.012716 |
| 32 | Protein | TSPAN1 | 0.139875 | 175 | Protein | LEG1 | 0.012146 |
| 33 | Protein | CGA | 0.137628 | 176 | Protein | THY1 | 0.011928 |
| 34 | Protein | CDON | 0.137580 | 177 | Protein | MMP3 | 0.011912 |
| 35 | Protein | CELSR2 | 0.135487 | 178 | Protein | PTN | 0.011850 |
| 36 | Protein | KLK4 | 0.132091 | 179 | Protein | MXRA8 | 0.011774 |
| 37 | Protein | WNT9A | 0.132053 | 180 | Protein | AGXT | 0.011489 |
| 38 | Protein | CA14 | 0.131972 | 181 | Protein | CLEC14A | 0.011376 |
| 39 | Protein | KLK14 | 0.114268 | 182 | Protein | CST1 | 0.011245 |
| 40 | Protein | ADAMTS16 | 0.109722 | 183 | Protein | SELL | 0.010891 |
| 41 | Protein | LECT2 | 0.109034 | 184 | Protein | HSD11B1 | 0.010843 |
| 42 | Protein | SOD2 | 0.108746 | 185 | Protein | PRSS2 | 0.010774 |
| 43 | Protein | COL6A3 | 0.103293 | 186 | Protein | EPHA4 | 0.010751 |
| 44 | Protein | CXCL9 | 0.100877 | 187 | Protein | BSG | 0.010407 |
| 45 | Protein | KIT | 0.098230 | 188 | Protein | NBL1 | 0.010360 |
| 46 | Protein | RRM2 | 0.097638 | 189 | Protein | ICAM4 | 0.010334 |
| 47 | Protein | NPPC | 0.097627 | 190 | Protein | SPINT1 | 0.010252 |
| 48 | Protein | IL15 | 0.097554 | 191 | Protein | AFAP1 | 0.009812 |
| 49 | Protein | CHIT1 | 0.097547 | 192 | Protein | BPIFB2 | 0.009804 |
| 50 | Protein | THBS2 | 0.096076 | 193 | Protein | CST6 | 0.009719 |
| 51 | Protein | PRL | 0.093718 | 194 | Protein | MYOC | 0.009626 |
| 52 | Protein | KLK7 | 0.092100 | 195 | Protein | FSTL1 | 0.009493 |
| 53 | Protein | AGER | 0.089721 | 196 | Protein | TCL1B | 0.009350 |
| 54 | Protein | NTF4 | 0.089311 | 197 | Protein | PTPRM | 0.009313 |
| 55 | Protein | CSF3 | 0.085989 | 198 | Protein | COL1A1 | 0.009282 |
| 56 | Protein | MEPE | 0.083170 | 199 | Protein | IFNLR1 | 0.009226 |
| 57 | Protein | GP2 | 0.082785 | 200 | Protein | CNTN3 | 0.009221 |
| 58 | Protein | CRTAC1 | 0.082490 | 201 | Protein | EPHB4 | 0.009204 |
| 59 | Protein | ACRV1 | 0.073647 | 202 | Protein | IKZF2 | 0.009189 |
| 60 | Protein | ITGB5 | 0.073298 | 203 | Protein | EFEMP1 | 0.009152 |
| 61 | Protein | PLAT | 0.070618 | 204 | Protein | CNTN5 | 0.009149 |
| 62 | Protein | ERBB4 | 0.069979 | 205 | Protein | CHI3L1 | 0.009095 |
| 63 | Protein | ANGPT2 | 0.067669 | 206 | Protein | TXLNA | 0.009073 |
| 64 | Protein | MEP1B | 0.064495 | 207 | Protein | DNAJB8 | 0.009070 |
| 65 | Protein | C1QL2 | 0.061479 | 208 | Protein | PRR4 | 0.008994 |
| 66 | Protein | CD1C | 0.057632 | 209 | Protein | MCAM | 0.008975 |
| 67 | Protein | ADGRG1 | 0.055589 | 210 | Protein | IL2RA | 0.008922 |
| 68 | Protein | COL4A1 | 0.054606 | 211 | Protein | APOC1 | 0.008900 |
| 69 | Protein | GIP | 0.052054 | 212 | Protein | NUDT2 | 0.008898 |
| 70 | Protein | FASLG | 0.051734 | 213 | Protein | ULBP2 | 0.008895 |
| 71 | Protein | COL9A1 | 0.051662 | 214 | Protein | VTCN1 | 0.008847 |
| 72 | Protein | GDF2 | 0.047884 | 215 | Protein | CCL20 | 0.008689 |
| 73 | Protein | GAS6 | 0.047177 | 216 | Protein | CXADR | 0.008494 |
| 74 | Protein | ENPP2 | 0.047152 | 217 | Protein | DKKL1 | 0.008331 |
| 75 | Protein | ROBO1 | 0.044233 | 218 | Protein | PRTN3 | 0.008246 |
| 76 | Protein | BGLAP | 0.044114 | 219 | Protein | PTPRZ1 | 0.008143 |
| 77 | Protein | RLN2 | 0.044017 | 220 | Protein | CX3CL1 | 0.008040 |
| 78 | Protein | CRH | 0.043488 | 221 | Protein | SDC1 | 0.007952 |
| 79 | Protein | WIF1 | 0.043013 | 222 | Protein | NECTIN4 | 0.007901 |
| 80 | Protein | COL15A1 | 0.042691 | 223 | Protein | KRT5 | 0.007838 |
| 81 | Protein | ITGAV | 0.042684 | 224 | Protein | FLT3 | 0.007783 |
| 82 | Protein | MOG | 0.042282 | 225 | Protein | ADGRE5 | 0.007722 |
| 83 | Protein | KIRREL2 | 0.039754 | 226 | Protein | CD63 | 0.007650 |
| 84 | Protein | CHRDL2 | 0.039175 | 227 | Protein | TGFA | 0.007569 |
| 85 | Protein | TNFSF13 | 0.039103 | 228 | Protein | CXCL13 | 0.007507 |
| 86 | Protein | CTHRC1 | 0.037940 | 229 | Protein | SELE | 0.007489 |
| 87 | Protein | PRELP | 0.037653 | 230 | Protein | LTA4H | 0.007471 |
| 88 | Protein | RGMB | 0.037239 | 231 | Protein | OLR1 | 0.007466 |
| 89 | Protein | PRDX6 | 0.036774 | 232 | Protein | HHEX | 0.007379 |
| 90 | Protein | SFRP4 | 0.036638 | 233 | Protein | MUC2 | 0.007368 |
| 91 | Protein | CD8A | 0.036482 | 234 | Protein | PLAUR | 0.007306 |
| 92 | Protein | NFASC | 0.036301 | 235 | Protein | TNR | 0.007287 |
| 93 | Protein | ANGPT1 | 0.035399 | 236 | Protein | RPL14 | 0.007256 |
| 94 | Protein | CDH2 | 0.034814 | 237 | Protein | CPA1 | 0.007255 |
| 95 | Protein | FGF5 | 0.034649 | 238 | Protein | CPB1 | 0.007254 |
| 96 | Protein | PROK1 | 0.032920 | 239 | Protein | PTGDS | 0.007241 |
| 97 | Protein | SEPTIN3 | 0.032334 | 240 | Protein | LXN | 0.007188 |
| 98 | Protein | SIT1 | 0.031823 | 241 | Protein | TSHB | 0.007176 |
| 99 | Protein | ISLR2 | 0.031723 | 242 | Protein | GPA33 | 0.007170 |
| 100 | Protein | GFRAL | 0.030819 | 243 | Protein | DLK1 | 0.007160 |
| 101 | Protein | NCAN | 0.029336 | 244 | Protein | F10 | 0.007126 |
| 102 | Protein | TNFRSF11B | 0.028263 | 245 | Protein | GHRL | 0.007027 |
| 103 | Protein | CYTL1 | 0.028094 | 246 | Protein | IGSF9 | 0.006920 |
| 104 | Protein | DCBLD2 | 0.027927 | 247 | Protein | RGS8 | 0.006884 |
| 105 | Protein | ITGBL1 | 0.026547 | 248 | Protein | ARTN | 0.006833 |
| 106 | Protein | ITGB2 | 0.026070 | 249 | Protein | IMMT | 0.006819 |
| 107 | Protein | CD93 | 0.026051 | 250 | Protein | AREG | 0.006780 |
| 108 | Protein | BCAN | 0.026018 | 251 | Protein | IL18RAP | 0.006710 |
| 109 | Protein | DSG4 | 0.025549 | 252 | Protein | SPINT3 | 0.006664 |
| 110 | Protein | CCN5 | 0.025548 | 253 | Protein | TYRO3 | 0.006660 |
| 111 | Protein | IL3RA | 0.025490 | 254 | Protein | CALB1 | 0.006597 |
| 112 | Protein | SPON2 | 0.024901 | 255 | Protein | TFRC | 0.006596 |
| 113 | Protein | IL17D | 0.024845 | 256 | Protein | ADAMTS13 | 0.006552 |
| 114 | Protein | SDK2 | 0.024708 | 257 | Protein | CDH15 | 0.006542 |
| 115 | Protein | SCG2 | 0.024575 | 258 | Protein | ART3 | 0.006519 |
| 116 | Protein | FBLN2 | 0.024091 | 259 | Protein | GCNT1 | 0.006489 |
| 117 | Protein | FABP4 | 0.022975 | 260 | Protein | XCL1 | 0.006402 |
| 118 | Protein | LMOD1 | 0.022970 | 261 | Protein | IL22RA1 | 0.006315 |
| 119 | Protein | CR2 | 0.022774 | 262 | Protein | PTPRF | 0.006300 |
| 120 | Protein | BAG3 | 0.022723 | 263 | Protein | PCOLCE | 0.006296 |
| 121 | Protein | PSG1 | 0.022699 | 264 | Protein | RBP2 | 0.006271 |
| 122 | Protein | NELL2 | 0.022582 | 265 | Protein | BRSK2 | 0.006265 |
| 123 | Protein | LPO | 0.021506 | 266 | Protein | CILP | 0.006239 |
| 124 | Protein | FABP9 | 0.021196 | 267 | Protein | DNPH1 | 0.006121 |
| 125 | Protein | SCLY | 0.020344 | 268 | Protein | NPTX2 | 0.006108 |
| 126 | Protein | CHRDL1 | 0.020233 | 269 | Protein | SEZ6L2 | 0.006101 |
| 127 | Protein | KLK6 | 0.020196 | 270 | Protein | TNFRSF4 | 0.006017 |
| 128 | Protein | NTRK3 | 0.020100 | 271 | Protein | AMBN | 0.005991 |
| 129 | Protein | ADGRE2 | 0.020065 | 272 | Protein | TNFAIP8 | 0.005927 |
| 130 | Protein | LILRB4 | 0.020050 | 273 | Protein | COMP | 0.005889 |
| 131 | Protein | PTPRN2 | 0.020033 | 274 | Protein | EGFR | 0.005880 |
| 132 | Protein | PINLYP | 0.019585 | 275 | Protein | CRX | 0.005835 |
| 133 | Protein | CCL11 | 0.019478 | 276 | Protein | TNF | 0.005805 |
| 134 | Protein | PKD1 | 0.019308 | 277 | Protein | TNFSF13B | 0.005772 |
| 135 | Protein | STC2 | 0.018413 | 278 | Protein | LAMA4 | 0.005759 |
| 136 | Protein | SKAP1 | 0.018142 | 279 | Protein | MSR1 | 0.005717 |
| 137 | Protein | PPY | 0.018096 | 280 | Protein | MYOM3 | 0.005687 |
| 138 | Protein | CD14 | 0.017931 | 281 | Protein | DAPP1 | 0.005648 |
| 139 | Protein | ENPP5 | 0.017511 | 282 | Protein | NFYA | 0.005580 |
| 140 | Protein | JUN | 0.017483 | 283 | Protein | APLP1 | 0.005573 |
| 141 | Protein | NTF3 | 0.017455 | 284 | Protein | ASGR1 | 0.005539 |
| 142 | Protein | CRYBB2 | 0.017454 | 285 | Protein | OMG | 0.005530 |
| 143 | Protein | LTA | 0.017175 | 286 | Protein | EPHA1 | 0.005514 |

| Rank | Type | Feature_Name | Importance |
| --- | --- | --- | --- |
| 1 | Metabolite | n-3 % | 0.114348 |
| 2 | Metabolite | DHA | 0.090507 |
| 3 | Metabolite | n-6/n-3 ratio | 0.083401 |
| 4 | Metabolite | n-3 FAs | 0.046949 |
| 5 | Metabolite | Glc–Lac | 0.041668 |
| 6 | Metabolite | ALB | 0.032737 |
| 7 | Metabolite | β-HB | 0.031123 |
| 8 | Metabolite | Glc | 0.017762 |
| 9 | Metabolite | Gly | 0.011577 |
| 10 | Metabolite | DHA % | 0.010529 |
| 11 | Metabolite | Cit | 0.010101 |
| 12 | Metabolite | DU | 0.008660 |
| 13 | Metabolite | PUFAs | 0.005976 |
| 14 | Metabolite | SFA % | 0.005860 |

* n-3 %: Omega-3 Fatty Acids to Total Fatty Acids percentage

n-6/n-3 ratio: Omega-6 Fatty Acids to Omega-3 Fatty Acids ratio

n-3 Fas: Omega-3 Fatty Acids

Glc–Lac: Glucose-lactate

DHA %: Docosahexaenoic Acid to Total Fatty Acids percentage

DU: Degree of Unsaturation

PUFAs: Polyunsaturated Fatty Acids

SFA %: Saturated Fatty Acids to Total Fatty Acids percentage

Supplemental Table 3: Ablation analysis of the StackAge ensemble framework

| Model | Pearson r | RMSE | MAE | R² |
| --- | --- | --- | --- | --- |
| StackAge(LR+EN+LGBM) | 0.927 | 3.018 | 2.37 | 0.859 |
| StackAge (EN+LGBM) | 0.901 | 3.358 | 2.598 | 0.816 |
| StackAge (LR+EN) | 0.861 | 4.099 | 3.239 | 0.74 |
| StackAge (LR+LGBM) | 0.838 | 4.396 | 3.472 | 0.701 |

Supplemental Table 4: Sensitivity analyses demonstrating that sex differences in aging rate are independent of chronological age

| Analysis | Statistics | Value | Interpretation |
| --- | --- | --- | --- |
| Correlation (aging rate vs age) | Pearson r | 0.0027 | No correlation |
|  | p-value | 0.644 | No evidence of association |
| Sex age difference (Female vs Male) | Mean age (Male) | 57.63 years | Negligible difference |
|  | Mean age (Female) | 57.27 years |  |
|  | Mean difference | 0.36 years |  |
|  | t-test p-value | 1.10 × 10⁻⁴ | Statistically significant but negligible effect size |
| ANCOVA (aging rate ~ sex + age) | Age effect p-value | 0.611 | No significant association |
|  | Sex effect p-value | 0.034 | Remains significant after adjustment |

Supplemental Table 5: Ablation analysis of StackAge performance after exclusion of disease-associated proteins

| Disease | r (Baseline) | r (Remove) | Δr | ΔRMSE |
| --- | --- | --- | --- | --- |
| IHD | 0.92701 | 0.92386 | -0.00315 | +0.06125 |
| Diabetes |  | 0.92576 | -0.00125 | +0.02397 |
| CKDs |  | 0.92553 | -0.00148 | +0.02886 |

Supplemental Table 6: Detailed results of KEGG pathway enrichment analysis for differentially expressed proteins

| Pathway | p-value | q-value | Prominent protein |
| --- | --- | --- | --- |
| Cytokine-cytokine receptor interaction | 0.0005 | 0.1127 | CXCL13; TNF; TNFSF13B; GDF15; GDF2; IL2RA; IL5RA; EDA2R; TNFRSF4 |
| ECM-receptor interaction | 0.0006 | 0.1127 | COL6A3; TNR; TNXB; HSPG2; ITGAV |
| Hematopoietic cell lineage | 0.0011 | 0.1276 | TNF; KIT; IL2RA; CD14; IL5RA |
| PI3K-Akt signaling pathway | 0.0019 | 0.1703 | PGF; EGFR; KIT; RET; IL2RA; COL6A3; TNR; TNXB; ITGAV |
| Focal adhesion | 0.005 | 0.2589 | COL6A3; TNR; TNXB; ITGAV; PGF; EGFR |
| Proteoglycans in cancer | 0.0051 | 0.2589 | WNT9A; ITGAV; IL2RA; IL5RA; PGF; EGFR; KIT; RET |
| Virion-Ebolavirus, Lyssavirus and Morbillivirus | 0.0038 | 0.2589 | ASGR1; NECTIN4 |
| Rheumatoid arthritis | 0.0064 | 0.2859 | ITGB2; TNFSF13B; TNF; MMP3 |
| Legionellosis | 0.0095 | 0.3378 | ITGB2; CD14; TNF |
| NF-kappa B signaling pathway | 0.0091 | 0.3378 | TNF; EDA2R; CD14; TNFSF13B |
| Regulation of lipolysis in adipocytes | 0.0109 | 0.3542 | TSHB; CGA; FABP4 |
| Human papillomavirus infection | 0.0149 | 0.4428 | WNT9A; COL6A3; TNR; TNXB; ITGAV; TNF; EGFR |
| Central carbon metabolism in cancer | 0.018 | 0.4931 | KIT; RET; EGFR |
| Thyroid hormone synthesis | 0.0208 | 0.5292 | TSHB; CGA; ASGR1 |
| Pertussis | 0.023 | 0.5474 | ITGB2; CD14; TNF |
| Hormone signaling | 0.0281 | 0.6181 | TSHB; CGA; FSHB; ITGAV; MLN |
| MAPK signaling pathway | 0.0294 | 0.6181 | PGF; EGFR; KIT; RET; TNF; CD14 |
| Phagosome | 0.0356 | 0.6488 | ITGB2; ITGAV; CD14; MSR1 |
| GnRH signaling pathway | 0.0361 | 0.6488 | CGA; FSHB; EGFR |
| Cell adhesion molecules | 0.0364 | 0.6488 | ITGB2; CDH2; ITGAV; NFASC |
